# Covariate-adjusted measures of discrimination for survival data

**DOI:** 10.1002/bimj.201400061

**Published:** 2014-12-20

**Authors:** Ian R. White, Eleni Rapsomaniki

**Affiliations:** 1 MRC Biostatistics Unit, Cambridge Institute of Public Health, Forvie Site, Robinson Way, Cambridge Biomedical Campus, Cambridge CB2 0SR, UK; 2 Farr Institute for Health Informatics Research, Department of Epidemiology and Public Health, University College London Medical School, 222 Euston Road, London WC1E 6BT, UK

**Keywords:** C-index, D-index, Discrimination

## Abstract

**Motivation:**

Discrimination statistics describe the ability of a survival model to assign higher risks to individuals who experience earlier events: examples are Harrell’s C-index and Royston and Sauerbrei’s D, which we call the D-index. Prognostic covariates whose distributions are controlled by the study design (e.g. age and sex) influence discrimination and can make it difficult to compare model discrimination between studies. Although covariate adjustment is a standard procedure for quantifying disease-risk factor associations, there are no covariate adjustment methods for discrimination statistics in censored survival data.

**Objective:**

To develop extensions of the C-index and D-index that describe the prognostic ability of a model adjusted for one or more covariate(s).

**Method:**

We define a covariate-adjusted C-index and D-index for censored survival data, propose several estimators, and investigate their performance in simulation studies and in data from a large individual participant data meta-analysis, the Emerging Risk Factors Collaboration.

**Results:**

The proposed methods perform well in simulations. In the Emerging Risk Factors Collaboration data, the age-adjusted C-index and D-index were substantially smaller than unadjusted values. The study-specific standard deviation of baseline age was strongly associated with the unadjusted C-index and D-index but not significantly associated with the age-adjusted indices.

**Conclusions:**

The proposed estimators improve meta-analysis comparisons, are easy to implement and give a more meaningful clinical interpretation.

## 1 Introduction

A fundamental property of a prognostic marker is its ability to discriminate high from low risk patients (Hlatky et al., 2009). Markers that do not improve discrimination are also unlikely to improve other measures of clinical performance (Mihaescu et al., 2010). The discrimination performance of a marker and its incremental value can vary significantly across different studies. Variation beyond chance can be attributed to differences between studies either in the strength of association between the marker and the outcome or in the marker’s distribution (Pepe et al., 2004), or to a range of other possible biases that relate to the conduct and recording in a study (Lijmer et al., 2002). In practice, markers are often used in combination, so interest lies in evaluating the discrimination of a prognostic model including one or more markers together with demographic variables. Other important aspects of prognostic ability include calibration.

For a binary outcome, the standard description of discrimination is the receiver operating characteristic (ROC) curve, which displays the trade-off between specificity (the probability that a control marker value is below the cut-off) and sensitivity (the probability that a case marker value is above the cut-off) for different marker cut-offs. The ROC curve is often summarized by the area under the curve (AUC), also called the C-statistic, which can be interpreted as the probability that the marker will correctly classify a randomly chosen pair of patients as case and noncase (Hanley and McNeil, 1982). The AUC ranges from 0 (when all predictions are wrong) and 1 (perfect predictions) with 0.5 representing the average discriminative ability of random predictions. Values below 0.5 are rarely seen other than due to small sample variation.

For a survival outcome, many measures of prognostic ability have been proposed (Choodari-Oskooei et al., 2012a, 2012b). The C-statistic can be used to measure discrimination in this setting, taking the binary outcome to be survival to a fixed follow-up time (Chambless and Diao, 2006). The C-index (Harrell et al., 1982) extends the C-statistic and avoids specifying a fixed follow-up time: it estimates the probability that given two randomly drawn patients, the patient who has an event first is predicted a higher risk. Royston and Sauerbrei (2004) proposed an alternative measure, D, which we call the D-index: it is based on a proportional hazards model and has the interpretation of an average log hazard ratio between an individual in the upper half of the risk distribution and an individual in the lower half (Pennells et al., 2014). We use the C-index because it is the most widely used measure in practice (Mallett et al., 2010), and the D-index because it adapts well to the purposes of this paper; the two measures give similar conclusions when used to evaluate the discrimination added by a new marker (Fibrinogen Studies Collaboration, 2009).

Covariate adjustment is necessary for correct assessment of disease-risk factor associations in observational studies, but its importance is rarely acknowledged in assessing discrimination. A particular issue is that covariates that form part of the study design such as age and sex can impact substantially on the prognostic ability of a model (Janes and Pepe, 2008; Kerr and Pepe, 2011): for example, the prognostic ability of a cardiovascular risk model (which includes age, sex, clinical covariates, and biomarkers) is likely to be substantially larger in a study that recruited men and women aged 40–80 than in a similar study that only recruited men aged 55–65. Janes and Pepe (2008), writing in the context of ROC curves, identify three cases in which covariates *Z* influence the discrimination performance of a risk score *R*:

1. The covariate *Z* is associated both with *R* and with disease risk. A common example is when *Z* is age. Janes and Pepe (2008) showthat stratifying by categorical*Z* reduces the discrimination of *R*. This reduction is greater if *R* and *Z* are highly correlated.
2. A different issue arises if the covariates *Z* are associated with *R* but not with disease risk. Ignoring this type of covariate effect may underestimate discrimination (Janes and Pepe, 2008). In cardiovascular disease, *R* might be a function of C-reactive protein, a cardiovascular risk factor, and *Z* might be acute infection, which strongly raises C-reactive protein. Allowing for *Z* in the analysis could remove a source of noise in *R* and hence improve discrimination.
3. The discrimination of *R* may vary across levels of *Z*, which is analogous to effect modification. This situation arises if the hazards associated with various levels of *Z* vary: for example, associations with blood pressure measurements are attenuated with increasing age (Prospective Studies Collaboration, 2002). It also arises when associations remain constant but the spread of the distribution of *X* varies with *Z*.

Covariate adjustment for measures of discrimination has been tackled in the context of diagnostic tests using ROC curves based on binary outcomes (Janes et al., 2009). However, there are currently no methods to adjust the discrimination performance of prognostic markers for covariates for censored survival data. In this paper, we propose definitions of the C-index and D-index for censored survival data allowing adjustment for one or more binary or continuous covariates, and ways to estimate them. Although we primarily aim to adjust for age and sex, the methods are presented for a general covariate adjustment.

The paper is arranged as follows. In Section 2 we describe the data which motivated our methods, and we define the model used to generate risk predictions. In Section 3, we review unadjusted measures of discrimination. In Section 4, we propose definitions of adjusted measures of discrimination, and our new estimators. In Section 5, we examine the performance of the proposed estimators in a simulation study. In Section 6 we apply the proposed methods to data from a large set of epidemiological cohort studies. We conclude in Section 7 with a discussion of our results, recommendations for when our proposed estimators might be appropriate, desirable extensions and limitations.

## 2 Data

The Emerging Risk Factors Collaboration (ERFC) has collated and harmonized individual participant data from population-based prospective studies of cardiovascular disease (CVD) (Emerging Risk Factors Collaboration, 2007). In May 2011 the data set comprised 1.9 million individuals in 108 studies with an average of 15.5 years follow-up. We used these data to model time to first fatal/nonfatal CVD event, which includes coronary heart disease and stroke. Our main dataset was restricted to prospective cohorts and clinical trials that provide information on Framingham risk factors, that is age, smoking status (current/ex vs. never), systolic blood pressure (SBP), total cholesterol (TC), high-density lipoprotein (HDL) cholesterol and history of diabetes at the baseline survey. Individual participant data were further restricted to subjects aged at least 40 years at baseline with the above risk factors recorded, no known history of CVD at the baseline survey, no recorded history of diabetes, and not known to be under statin treatment. Thus, our analysis data comprised 349,137 individuals from 82 studies (of which eight were clinical trials), of whom 24,369 experienced a CVD event.

The model fitted to these data was the Cox proportional hazards model stratified by study and sex. Studies that were randomized trials were additionally stratified by trial arm. Thus, for individual *i* in stratum *s*, the hazard at time *t* is

(1) $$h_{si}(t)=h_{0s}(t)\exp\;(\beta x_{i}),$$

where **x***_i_* is the vector of covariates (age, smoking status, SBP, TC, and HDL cholesterol) for individual *i*, **β** is a vector of corresponding regression coefficients (assumed constant across strata), and *h*_0_*_s_*(*t*) is the baseline hazard at time *t* for individuals of stratum *s*. Table 1 summarizes the data from these 82 studies and the fitted Cox model.

**Figure 1:**
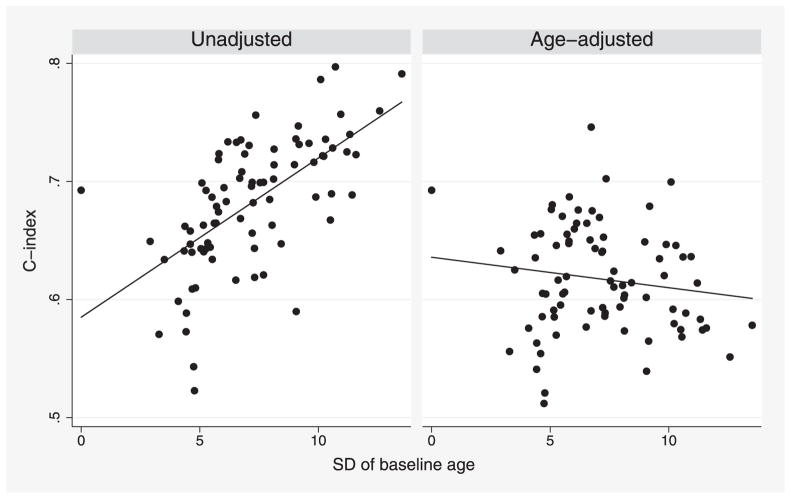
ERFC data: unadjusted and age-adjusted C-index for a model including baseline age, smoking, SBP, TC, and HDL, plotted for each study against the SD of baseline age in that study. Analyses are stratified by sex and trial arm. Each point represents one study.

The within-study distribution of baseline age and sex is determined by a study design and hence differs between studies. This may affect measures of discrimination. Model (1) is stratified by sex, so standard calculations automatically stratify measures of discrimination for sex. We therefore focus on the effect of baseline age. The left-hand panel in Fig. 1 plots the C-index for each study (computed as described in Section 6) against the within-study standard deviation (SD) of baseline age. Studies with more variation in baseline age tend to have substantially larger C-indices. The age-adjusted C-index, introduced in Section 4 below, is plotted in the right-hand panel, and shows no association with variation in baseline age.

## 3 Measures of discrimination in the absence of covariate adjustment

### 3.1 Notation

We initially work in a single dataset of *n* individuals. For each individual *i* = 1*,*…*, n*, we assume that the model covariates are **x***_i_* (scalar or vector), the true event time (in the absence of censoring) is $t_{i}^{*}$, and censoring time is *c_i_*, so the event indicator is $d_{i}=1(t_{i}^{*}\leq c_{i})$ and the observed time is $t_{i}=\min\;(t_{i}^{*},c_{i})$. The observed data are (*x_i_, d_i_, t_i_*). Censoring is assumed to be noninformative.

We also assume that the risk score is (scalar) *r*(**x***_i_*), which is typically (but not necessarily) the linear predictor **β̂***_x_***x** from fitting a survival model such as *h*(*t*) = *h*_0_(*t*) exp (**β***_x_***x**) where **β***_x_* are coefficients and *h*_0_(*t*) is the baseline hazard. Our aim is to evaluate the discrimination of *r*(**x***_i_*).

### 3.2 C-index

Harrell et al. (1982) defined the C-index as a statistic measuring the degree to which sample pairs are concordant, where concordance occurs if the individual of higher predicted risk has the first event in the pair. This statistic is affected by censoring (see Section 3.2.1). The underlying estimand was defined by Heagerty and Zheng (2005) and Uno et al. (2011) as $C=P(r(x_{i})>r(x_{j})|t_{i}^{*}<t_{j}^{*})$. Gonen and Heller (2005) instead stated the estimand $K=P(t_{i}^{*}<t_{j}^{*}|r(x_{i})\geq r(x_{j}))$.

These estimands are equivalent in the absence of ties in *r*(**x***_i_*) (i.e., if all individuals have different values of *r*(**x***_i_*)). We believe that ties in *r*(**x***_i_*) are important, since poorly discriminating models may have many ties, so it is important to account for them. Heagerty and Zheng’s *C* counts pairs tied on *r*(**x***_i_*) as discordant, while *K* double-counts them (because they satisfy both *r*(**x***_i_*) ≥ *r*(**x***_j_*) and *r*(**x***_j_*) ≥ *r*(**x***_i_*)). Instead, we make the natural definition of the C-index as

(2) $$C=E\;\left[C_{ij}\right]\;\text{where}\;C_{ij}=\left\{ \begin{array}{cc} 1(t_{i}^{*}<t_{j}^{*}) & \text{if}\;r(x_{i})>r(x_{j})\\ 0.5 & \text{if}\;r(x_{i})=r(x_{j})\\ 1(t_{i}^{*}>t_{j}^{*}) & \text{if}\;r(x_{i})<r(x_{j}) \end{array}\right.$$

for a random pair (*i, j*), where 1(*a*) = 1 if *a* is true and 0 if *a* is false.

Pairs with tied event times are excluded from all calculations based on *C_ij_*, so that the estimand becomes $E[C_{ij}|t_{i}^{*}\neq t_{j}^{*}]$. For simplicity, however, we ignore tied event times in the notation throughout this article.

We now consider various estimators of *C* in Eq. (2).

#### 3.2.1 Harrell’s estimator

Estimation of *C* is complicated by the presence of censoring, because we do not know whether $t_{i}^{*}<t_{j}^{*}$ for pairs where the first event time is censored. Harrell et al. (1996) proposed estimating *C* as the mean of *C_ij_* over informative pairs, where pair (*i, j*) is informative if $t_{i}^{*}<t_{j}^{*}$ and *d_i_* = 1 or $t_{i}^{*}>t_{j}^{*}$ and *d_j_* = 1: that is, if the first event in the pair is observed. Harrell’s estimator is often written as

$${\hat{C}}_{\mathrm{Har}}=\frac{\#\text{concordant}+\frac{1}{2}\#\text{tied}}{\#\text{concordant}+\#\text{discordant}+\#\text{tied}}$$

where #concordant counts pairs with $t_{i}^{*}<t_{j}^{*}$ and *r*(**x***_i_*) *> r*(**x***_j_*), or $t_{i}^{*}>t_{j}^{*}$ and *r*(**x***_i_*) *< r*(**x***_j_*); #tied counts pairs with *r*(**x***_i_*) = *r*(**x***_j_*); and #discordant counts pairs with $t_{i}^{*}<t_{j}^{*}$ and *r*(**x***_i_*) *< r*(**x***_j_*), or $t_{i}^{*}>t_{j}^{*}$ and *r*(**x***_i_*) *> r*(**x***_j_*). However, the informative pairs are not representative of all pairs—for example, a pair of low-risk individuals is likely to have no event and hence be noninformative—and this can cause bias in *Ĉ_Har_* (Gonen and Heller, 2005).

#### 3.2.2 Gonen and Heller’s estimator

Gonen and Heller (2005) proposed an alternative estimator to avoid bias due to censoring. To present the idea in greater generality, suppose r^*^(**x***_i_*) is a linear predictor from a correctly specified proportional hazards model. Then r^*^(**x***_i_*) − r^*^(**x***_j_*) represents the log hazard ratio between individuals *i* and *j*, and the probability that individuals *i* and *j* are concordant is expit {r^*^(**x***_i_*) − r^*^(**x***_j_*)} if *r*(**x***_i_*) *> r*(**x***_j_*) where expit(*η*) = 1*/*(1 + exp (−*η*)). (Similarly it is expit {r^*^(**x***_j_*) − r^*^(**x***_i_*)} if *r*(**x***_i_*) *< r*(**x***_j_*), and 0.5 if *r*(**x***_i_*) = *r*(**x***_j_*)). Then the estimator is the average of this concordance probability, which can be written as

(3) $${\hat{C}}_{\mathrm{ind}}=\frac{1}{n(n-1)}\sum_{i,j}\text{expit}\;\left\{[r^{*}(x_{i})-r^{*}(x_{j})]\text{sign}\;\left[r(x_{i})-r(x_{j})\right]\right\}.$$

Gonen and Heller (2005) considered the special case r^*^(**x***_i_*) = *r*(**x***_i_*) (that is, they assumed that *r*(**x***_i_*) is a linear predictor from a correctly specified proportional hazards model) giving the simpler expression

(4) $${\hat{C}}_{\mathrm{ind}}=\frac{1}{n(n-1)}\sum_{i,j}\text{expit}\left(|r(x_{i})-r(x_{j})|\right).$$

*Ĉ_ind_* is an indirect measure, since it does not use the event times and relies on correct model specification.

#### 3.2.3 Restricted C-index

Let *τ* = max*_i_ t_i_* be the longest follow-up time observed. The study only gives information about discrimination at time *t* ≤ *τ*, and *C* can only be estimated by (implicitly) extrapolating to times *t > τ*: for example, *Ĉ_ind_* assumes that the proportional hazards model continues to hold at times beyond *τ*. To avoid extrapolation, Heagerty and Zheng (2005) proposed the restricted C-index

$$C^{\tau }=P(r(x_{i})>r(x_{j})|t_{i}^{*}<t_{j}^{*},t_{i}^{*}<\tau )$$

which is estimable without extrapolation in a study with follow-up at least up to time *τ*. They and Uno et al. (2011) proposed estimators of *C^τ^* to account for censoring before time *τ* : that of Uno et al. (2011) involves a weighted mean of *C_ij_* over informative pairs, where the weight for pair (*i, j*) is *Ĝ*(min (*t_i_, t_j_*))^−2^ and *G*(*t*) = *P*(*c_i_* ≥ *t*).

### 3.3 D-index

Royston and Sauerbrei (2004) proposed a measure, *D*, which we also call the D-index, with the interpretation of the log hazard ratio between two equal-sized prognostic groups. It is estimated in a two-stage procedure. In stage 1, the values of *r*(**x***_i_*) are ranked, converted to normal scores, and multiplied by $\sqrt{\pi /8}$. In stage 2, a proportional hazards regression is performed on the scaled normal scores, and *D* is the regression coefficient.

As in the work of Harrell et al. (1982), the estimand is not immediately clear. A possible estimand is based on pairs: still assuming that r^*^(**x***_i_*) − r^*^(**x***_j_*) is the true log hazard ratio between individuals *i* and *j*, the estimand *D* can be defined as the average of this log hazard ratio when *i* is drawn randomly from the upper half of the risk distribution and *j* is drawn randomly from the lower half (Pennells et al., 2014): that is,

(5) $$D=E\;\left[r^{*}(x_{i})-r^{*}(x_{j})|r(x_{i})>\bar{r}>r(x_{j})\right]$$

where *r̄* is the mean of the *r*(**x***_i_*). The algorithm above clearly estimates this estimand consistently when the proportional hazards model is correctly specified and *r*(**x***_i_*) is normally distributed, but it may be biased when *r*(**x***_i_*) is skewed (Choodari-Oskooei et al., 2012a).

## 4 Measures of discrimination with covariate adjustment

Let **z***_i_* be covariates, which may or may not form part of **x***_i_*. We aim to evaluate the risk score *r*(**x***_i_*) while adjusting for the covariates **z***_i_*. Conceptually, we want to estimate *C* and *D* if we had a sample with a common value of *z*, or by restricting attention to pairs with equal values of *z*. In the ERFC data of Section 2, *r*(**x***_i_*) is a cardiovascular risk prediction while *z_i_* is age.

### 4.1 Adjusted *C*

#### 4.1.1 Estimand

Covariate adjustment can be defined by considering pairs that match exactly on *Z*, so that

$$C(z)=E\;\left[C_{ij}|z_{i}=z_{j}=z\right]$$

is a *z*-specific *C*. For situations where *C*(*z*) is roughly constant over *z*, or where a summary measure of discrimination is required, the *z*-adjusted *C*

$$C^{\mathrm{adj}}=E\;\left[C_{ij}|z_{i}=z_{j}\right]$$

is the natural measure when a risk model is stratified by *z*, and can be applied more widely.

Note that for continuous *z* with density *f*(*z*), pairs matching on *z* have density proportional to *f*(*z*)^2^, so that

(6) $$C^{\mathrm{adj}}=\int C(z)f{\left(z\right)}^{2}dz\int f{\left(z\right)}^{2}dz.$$

It is natural to consider weighting by *f*(*z*) rather than *f*(*z*)^2^ in (6), so we also define

$$C^{\mathrm{adj},w}=\int C(z)f(z)dz\int f(z)dz$$

although other choices of weights are also possible. Of course, *C^adj,w^* = *C^adj^* if *C*(*z*) is constant.

#### 4.1.2 Direct estimation for categorical Z

We describe an estimator as direct (like *Ĉ_Har_*) if it uses actual event times, and indirect (like *Ĉ_ind_*) if instead it uses risks predicted under a model. Direct estimation is tricky with continuous *z*, as there may be few or no matching pairs (Section 4.1.3). We therefore first consider the case with categorical *z*. Simple estimators are *Ĉ*(*z*) = {Σ_(*i,j*):*z*_*i*_=*z*_*j*_=*z*_*C*_*ij*_}*/*{Σ_(*i,j*):*z*_*i*_=*z*_*j*_=*z*_ 1}, *Ĉ^adj^* = {Σ_(*i,j*):*z*_*i*_=*z*_*j*__ *C_ij_*}*/*{Σ_(*i, j*):*z*_*i*_=*z*_*j*__ 1} and *Ĉ^ad j,w^* = {Σ*_z_ f̂*(*z*)*Ĉ*(*z*)}*/*{Σ*_z_ f̂*(*z*)}*,* where *f̂*(*z*) = Σ_*i:z*_*i*_=*z*_ 1. In the presence of censoring, these sums are restricted to informative pairs, and the weighting scheme of Uno et al. (2011) may be used to handle random censoring.

#### 4.1.3 Direct estimation for continuous or multivariate Z

For some methods, it is helpful to decompose

(7) $$r(x_{i})=m(x_{i},z_{i})+\hat{r}(z_{i})$$

where *m*(**x***_i_, z_i_*) is uncorrelated with *z_i_*. This is easily done by fitting a suitable regression for *r*(**x***_i_*) on *z_i_*, and defining *r̂*(*z_i_*) = E[*r*(**x***_i_*)|*z_i_*] as the fitted value and *m*(**x***_i_, z_i_*) as the residual. Conceptually, we want to estimate the discrimination that is due to *m*(**x***_i_, z_i_*). The methods proposed in this section do not assume that *m*(**x***_i_, z_i_*) is independent of *z_i_*, unlike the methods proposed in Section 4.1.4.

We propose direct estimation by plotting *C_ij_* against *r̂*(*z_j_*) − *r̂*(*z_i_*) or |*r̂*(*z_j_*) − *r̂*(*z_i_*)|, fitting a suitable model (parametric or nonparametric), and taking ${\hat{C}}_{\mathrm{smooth}1}^{\mathrm{adj}}$ as the fitted value at *r̂*(*z_j_*) − *r̂*(*z_i_*) = 0. In order to automate the procedure, we use a logistic regression of *C_ij_* on (*r̂*(*z_j_*) − *r̂*(*z_i_*))^2^ with weights

(8) $$w_{1}(z_{i},z_{j})=\exp\;\left(-\lambda {\left[\hat{r}(z_{j})-\hat{r}(z_{i})\right]}^{2}\right)$$

where *λ* controls the amount of smoothing. ${\hat{C}}_{\mathrm{smooth}1}^{\mathrm{adj}}$ is then the inverse logit of the estimated intercept. The procedure is illustrated in Supporting Information Fig. S1.

Again, in the presence of censoring, the sums are restricted to informative pairs, and the weighting scheme of Uno et al. (2011) may be used to handle random censoring.

We estimate *Ĉ^adj,w^* using the same logistic regression but with weights *w*_1_(*z_i_, z_j_*)*w*_2_(*z_i_, z_j_*) where

(9) $$w_{2}(z_{i},z_{j})={\left\{\hat{f}(z_{i})\hat{f}(z_{j})\right\}}^{-1/2}$$

since this weight approximates 1*/f* (*z*) when *z_i_* ≈ *z_j_*. Here, *f̂*(*z*) might be a kernel estimate of the density of *z*.

The above method is based on *C_ij_* which represents whether two events occur in the order predicted by *r*(**x**). An alternative is to explore whether events occur in the order predicted by *m*(**x***, z*). We define “m-concordance” $C_{ij}^{m}$ by replacing conditions *r*(**x***_i_*) *> r*(**x***_j_*) etc. in Eq. (2) with *m*(**x***_i_, z_i_*) *> m*(**x***_j_, z_j_*) etc. Again, fitted values of the mean of $C_{ij}^{m}$ at *r̂*(*z_j_*) − *r̂*(*z_i_*) = 0 give an estimator of *Ĉ^adj^*, which we denote by ${\hat{C}}_{\mathrm{smooth}2}^{\mathrm{adj}}$.

Comparing the two estimators ${\hat{C}}_{\mathrm{smooth}1}^{\mathrm{adj}}$ and ${\hat{C}}_{\mathrm{smooth}2}^{\mathrm{adj}}$ may help to detect an unsuitable value of *λ*. Too small a value causes bias by giving too much weight to mismatched pairs, while too large a value causes large variance by reducing the effective number of pairs used. We used the ERFC data to compare ${\hat{C}}_{\mathrm{smooth}1}^{\mathrm{adj}}$ with ${\hat{C}}_{\mathrm{smooth}2}^{\mathrm{adj}}$ (Supporting Information Fig. S2) and to compare their standard errors (Supporting Information Fig. S3), for 0 ≤ *λ* ≤ 10. Values *λ <* 1 tended to give large differences between the two estimators, but values in the range 1–10 seemed broadly reasonable: later work uses *λ* = 3.

#### 4.1.4 Indirect estimation of an approximate estimand

Now we use the correctly specified linear predictor r^*^(**x**), which we decompose as r^*^(**x***_i_*) = m^*^(**x***_i_, z_i_*) + r̂^*^(*z_i_*) as in (7). Recall that *P*(*C_ij_* = 1|**x***_i_,* **x***_j_*) = expit {r^*^(**x***_i_*) − r^*^(**x***_j_*)} when *r*(**x***_i_*) *> r*(**x***_j_*), etc. Hence for pairs that match on *z*, *P*(*C_ij_* = 1|**x***_i_,* **x***_j_*) = expit (m^*^(**x***_i_, z_i_*) − m^*^(**x***_j_, z_j_*)) when *m*(**x***_i_, z_i_*) *> m*(**x***_j_, z_j_*), etc. This suggests defining a new estimand

(10) $$C^{\mathrm{adj}*}=E\;\left[\text{expit}\;\left\{[m^{*}(x_{i},z_{i})-m^{*}(x_{j},z_{j})]\;\text{sign}\;\left[m(x_{i},z_{i})-m(x_{j},z_{j})\right]\right\}\right].$$

C^adj^^*^ = *C^adj^* if *m*(**x***_i_, z_i_*) is independent of *z_i_*. Appendix B and the simulation study demonstrate that C^adj^^*^ ≠ *C^adj^* in general, but differences are not large.

Analogous to (3), we propose the indirect estimator in the correctly specified case m^*^(**x***, z*) = *m*(**x***, z*):

(11) $${\hat{C}}_{\mathrm{ind}}^{\mathrm{adj}*}=\frac{1}{n(n-1)}\sum_{i,j}\text{expit}\;\left(|m(x_{i},z_{i})-m(x_{j},z_{j})|\right).$$

Like *Ĉ_ind_*, this estimator is unaffected by censoring, but requires correct model specification.

#### 4.1.5 Recalibrating

To be useful in practice, a risk score must be well calibrated. Ideally, this is ensured by recalibrating the model in an external validation set. However, sometimes miscalibrated risk scores are evaluated, and in this case we want to be sure that the miscalibration does not distort the C-index.

The advantage of a direct method is that it should give correct results if the risk score is miscalibrated. The indirect methods above are very susceptible to miscalibration. However, even the direct methods of Section 4.1.3 are slightly affected by miscalibration, because the weights in (8) are affected. We therefore propose preceding all the above methods, except for Harrell’s method (which is unaffected by miscalibration), by a recalibration step.

For the unadjusted indirect method, we assume r^*^(**x**) = *γ_r_r*(**x**) and estimate *γ_r_* by fitting the Cox model

$$h_{i}(t)=h_{0}(t)\exp\;\left\{\gamma_{r}r(x_{i})\right\}.$$

If *r*(**x**) is well calibrated, then *γ̂_r_* ≈ 1. The recalibrated estimate is

(12) $${\hat{C}}_{\mathrm{ind},\mathrm{recal}}=\frac{1}{n(n-1)}\sum_{i,j}\text{expit}\;\left({\hat{\gamma }}_{r}|r(x_{i})-r(x_{j})|\right).$$

For the adjusted methods, we assume m^*^(**x**, *z*) = *γ_m_m*(**x**, *z*) and r̂^*^(**x**) = *γ_r̂_r̂*(**x**) and estimate *γ_m_* and *γ_z_* by fitting the Cox model

$$h_{i}(t)=h_{0}(t)\exp\;\left\{\gamma_{m}m(x_{i},z_{i})+\gamma_{z}\hat{r}(z_{i})\right\}$$

with fixed *m*(**x***_i_*, *z_i_*) and *r̂*(*z_i_*). The recalibrated estimate is

(13) $${\hat{C}}_{\mathrm{ind},\mathrm{recal}}^{\mathrm{adj}*}=\frac{1}{n(n-1)}\sum_{i,j}\text{expit}\;\left({\hat{\gamma }}_{m}|m(x_{i},z_{i})-m(x_{j},z_{j})|\right).$$

Definitions (12) and (13) allow for negative values of *γ̂_r_* and *γ̂_m_*, which could arise with a very poorly calibrated model, and would correctly give estimates less than 0.5.

If *r*(**x**) is the linear predictor from fitting a Cox model to the data, then recalibration as proposed above is pointless: if done, it yields *γ̂_m_* = *γ̂_z_* = 1. However, the values of *γ_r_* and *γ_m_* in (12) and (13) could instead be estimated by shrinkage methods (Copas, 1983; van Houwelingen and Le Cessie, 1990).

### 4.2 Adjusted *D*

We define covariate-adjusted *D* by extending estimand (5) proposed in Section 3. First, *z*-specific *D* is

$$D(z)=E\;\left[r^{*}(x_{i})-r^{*}(x_{j})|r(x_{i})>\hat{r}(z)>r(x_{j}),z_{i}=z_{j}=z\right]$$

recalling that *r̂*(*z*) is the *z*-specific mean of the *r*(**x***_i_*). We can also write *D*(*z*) as

$$E\;\left[m^{*}(x_{i},z_{i})-m^{*}(x_{j},z_{j})|m(x_{i},z_{i})>0>m(x_{j},z_{j}),z_{i}=z_{j}=z\right]$$

and so it is natural to define adjusted *D* as

$$D^{\mathrm{adj}}=E\;\left[m^{*}(x_{i},z_{i})-m^{*}(x_{j},z_{j})|m(x_{i},z_{i})>0>m(x_{j},z_{j}),z_{i}=z_{j}\right].$$

That is, *D^adj^* is the average log hazard ratio between individuals matched on *z* who are above-average and below-average for their value of *z*.

We propose the following modification to the estimation algorithm for *D^adj^* given in Section 3.3. In stage 1, instead of ranking the *r*(**x***_i_*), we rank the *m*(**x***_i_*, *z_i_*) across the whole sample, form normal scores, and scale by $\sqrt{\pi /8}$. In stage 2, the proportional hazards regression on the scaled normal scores is adjusted for *z* to avoid bias from omitting a prognostic covariate (Ford et al., 1995). *D̂^adj^* is the coefficient of the scaled normal scores in the stage 2 model.

### 4.3 Stratification

A stratified version of *C^adj^* may be computed by restricting attention to pairs within strata. For stratified versions of C^adj^^*^ and *D^adj^* we replace *m*(**x***_i_*, *z_i_*) and *r̂*(*z_i_*) above with *m*(**x***_i_*, *z_i_*, *s_i_*) and *r̂*(*z_i_*, *s_i_*) where *s_i_* is the stratum of individual *i*, *r̂*(*z_i_*, *s_i_*) = E[*r*(**x***_i_*)|*z_i_*, *s_i_*] and *r*(**x***_i_*, *s_i_*) = *m*(**x***_i_*, *z_i_*, *s_i_*) + *r̂*(*z_i_*, *s_i_*). This decomposition is performed by regressing *r*(**x***_i_*, *s_i_*) on *z_i_* within strata. In estimating *D^adj^*, the stage 2 proportional hazards regression is stratified by *s*.

## 5 Simulation study

We next explore the performance (bias and precision) of the proposed estimators as we vary the strengths of association of the outcome with **x** and *z*. We first consider an ideal setting where *r*(**x**) is the linear predictor from a correctly specified Cox model, and *m*(**x***_i_*, *z_i_*) is independent of *z_i_* so that *C^adj^* = C^adj^^*^. We then consider a nonideal setting where var(*m*(**x***_i_*, *z_i_*)) depends on *z_i_*, so that estimands *C^adj^*, *C^adj^*^,^*^w^*, and C^adj^^*^ potentially differ.

### 5.1 Data generating model

Data sets of size *n* = 1000 were generated with covariates **x** = (*v*, *z*). This relatively large sample size was designed to make optimism negligible without excessively increasing computing time for *C* (which is roughly proportional to *n*^2^).

Covariate *z* represents age at baseline. A fraction 1 − *ϕ* of individuals belong to age group 1 and have *z* ~ *U*(40, 50), the uniform distribution from 40 to 50. The remaining fraction *ϕ* of individuals belong to age group 2 and have *z* ~ *U*(50, 60). Covariate *v* represents the biomarker of interest and was drawn as *v* = *α*(*z* − 50) + *u* with $u\sim N(0,\sigma_{g}^{2})$ for an individual whose value of *z* places them in age group *g*. Settings for *σ_g_* are given below. We chose *α* to make corr(*v*, *z*) = 0.25: changing corr(*v*, *z*) to 0 or 0.5 affected the unadjusted results for both C and D, but had negligible effect on the adjusted results (results not shown).

Survival times were drawn from the Gompertz distribution

$$h(t)=h_{0}\exp\;\{\beta_{v}v+\beta_{z}(z-50+t)\}$$

where *t* is time in years from baseline. We took *β_v_* = 0, 0.5, 1, and *β_z_* = 0, 0.1, 0.2. Follow-up was for 15 years, and *h*_0_ was chosen to give 50–70% censoring. With this data generating model, *r*(**x**) = *β_v_v* + *β_z_*(*z* − 50), *r̂*(*z*) = (*β_v_α* + *β_z_*)(*z* − 50) and *m*(**x**, *z*) = *β_v_*[*v* − *α*(*z* − 50)].

In simulation 1, we take *ϕ* = 0.5, so that *z* ~ *U*(40, 60) and weighting does not affect the estimands. We also take *σ*_1_ = *σ*_2_ = 1, so that *r̂*(*z*) and *m*(**x**, *z*) are independent, and the estimands *C^adj^* and C^adj^^*^ are equal. In simulation 2, we take *ϕ* = 2/3, in order to explore weighting, and *σ*_1_ = 1 and *σ*_2_ = 2, so that var(*m*(**x**, *z*)|*z*) depends on *z* and the estimands differ.

### 5.2 Methods considered

For *C*, the unadjusted methods considered were Harrell’s *Ĉ_Har_* (“Harrell”) and Gonen and Heller’s *Ĉ_ind_* (“indirect”). The adjusted methods considered were ${\hat{C}}_{\mathrm{smooth}1}^{\mathrm{adj}}$ (“smooth 1 unweighted”) and ${\hat{C}}_{\mathrm{smooth}2}^{\mathrm{adj}}$ (“smooth 2 unweighted”) with weights *w*_1_(*z_i_*, *z_j_*); ${\hat{C}}_{\mathrm{smooth}1}^{\mathrm{adj},w}$ (“smooth 1 weighted”) and ${\hat{C}}_{\mathrm{smooth}2}^{\mathrm{adj},w}$ (“smooth 2 weighted”) with weights *w*_1_(*z_i_*, *z_j_*)*w*_2_(*z_i_*, *z_j_*); and ${\hat{C}}_{\mathrm{ind}}^{\mathrm{adj}*}$ (“indirect”). Weight *w*_1_(*z_i_*, *z_j_*) was computed using (8) with *λ* = 3, and *w*_2_(*z_i_*, *z_j_*) was computed using (9) and estimating *f̂*(*z*) in one-unit bins for *z*. Estimation was restricted to informative pairs without the weighting of Uno et al. (2011).

For *D*, we used unadjusted *D̂* and adjusted *D̂^adj^*. The methods are summarized in Table 2 and the top half of Table 3.

### 5.3 Simulation scheme

A total of 1000 datasets were drawn for each combination of parameters. For each combination of parameters and each method, we computed *C̄* and *s_C_*, the mean and standard deviation of *Ĉ*, and we present a forest-type plot showing *C̄* with an interval constructed as *C̄* ± 1.96*s_C_*. We computed the true values of *C* and *D* using the exact methods described in appendices C and D. Source code to reproduce the results is available as Supporting Information on the journal’s web page (http://onlinelibrary.wiley.com/doi/10.1002/bimj.201400061/suppinfo).

### 5.4 Results for simulation 1

**Figure 2:**
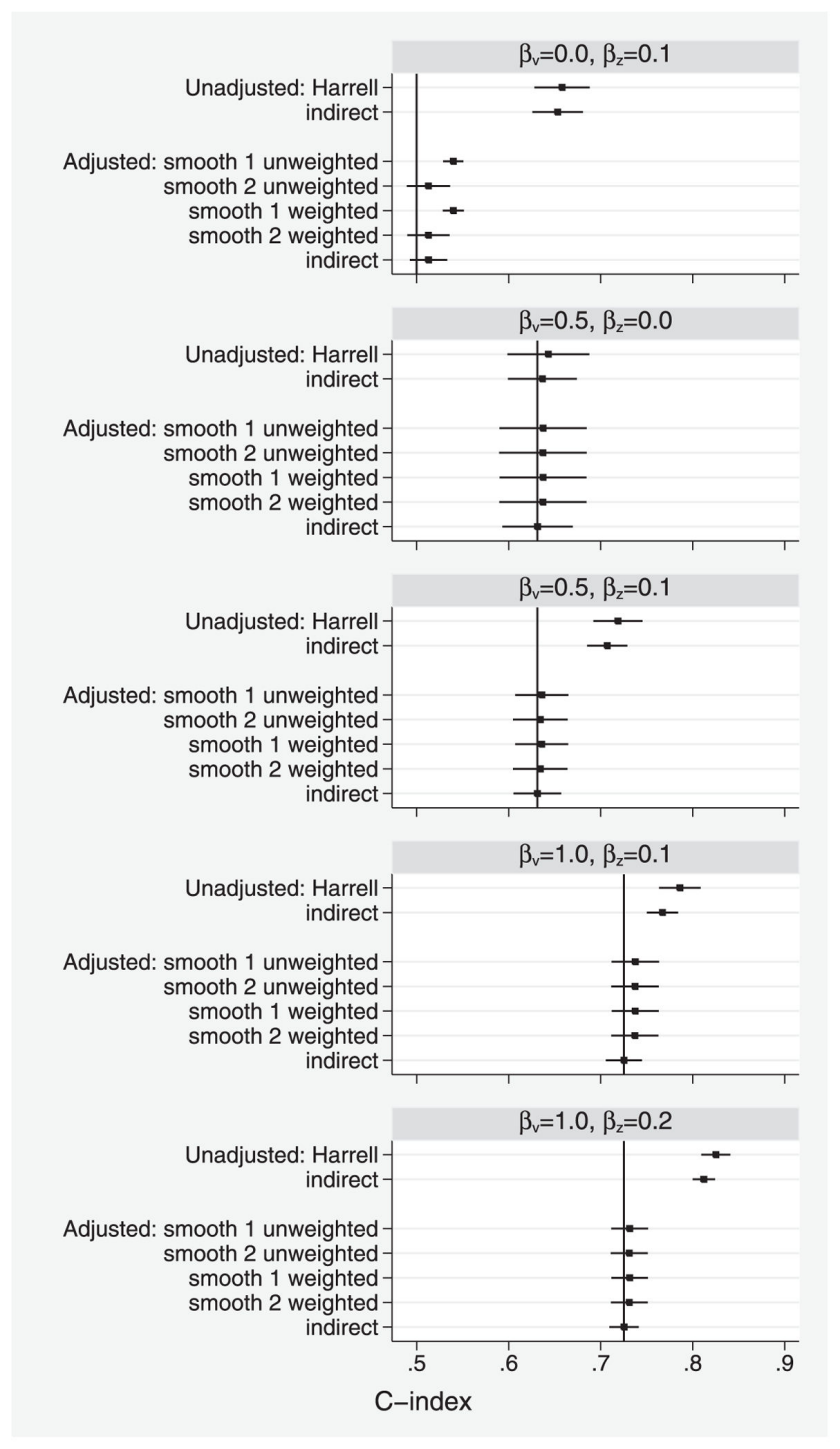
Simulation study 1: comparison of various unadjusted and adjusted estimates of *C*. Intervals show *C̄* ± 1.96*s_C_* where *C̄* and *s_C_* are the mean and standard deviation of *Ĉ*. Vertical lines indicate the true value of adjusted *C*. Panels show simulated data with different values of *β_v_* and *β_z_*, but all have corr(*v*, *z*) = 0.25 (see text).

Figure 2 shows results with corr(*v*, *z*) = 0.25: the five panels show different combinations of *β_v_* and *β_z_*.

Considering the unadjusted results, *Ĉ_Har_* has a slightly larger mean than *Ĉ_ind_* in all panels, indicating small bias due to censoring.

Comparing the unadjusted and adjusted estimates, we see that they are similar in the second panel where *β_z_* = 0 (i.e., where there is no covariate effect to adjust for), but markedly different in the other panels where *β_z_* > 0.

We now compare the different adjustment methods in the first panel where *β_v_* = 0 so that the true value is *C^adj^* = 0.5. The “smooth 1” methods (unweighted and weighted) show substantial bias. This is likely to have arisen because concordance *C_ij_* is strongly related to *r̂*(*z_j_*) − *r̂*(*z_i_*) and the smoothing method inadequately allows for this association. The other adjusted methods show small positive bias. This is attributable to optimism, since the models are fitted and evaluated in the same data; however, optimism is small because of our large sample size. We therefore suggest that “smooth 2” may be preferable to “smooth 1”.

In the other panels where *C^adj^* > 0.5, the indirect estimator appeared unbiased (suggesting the bias from optimism is negligible), while the smoothing estimators had small positive bias, presumably due to censoring.

**Figure 3:**
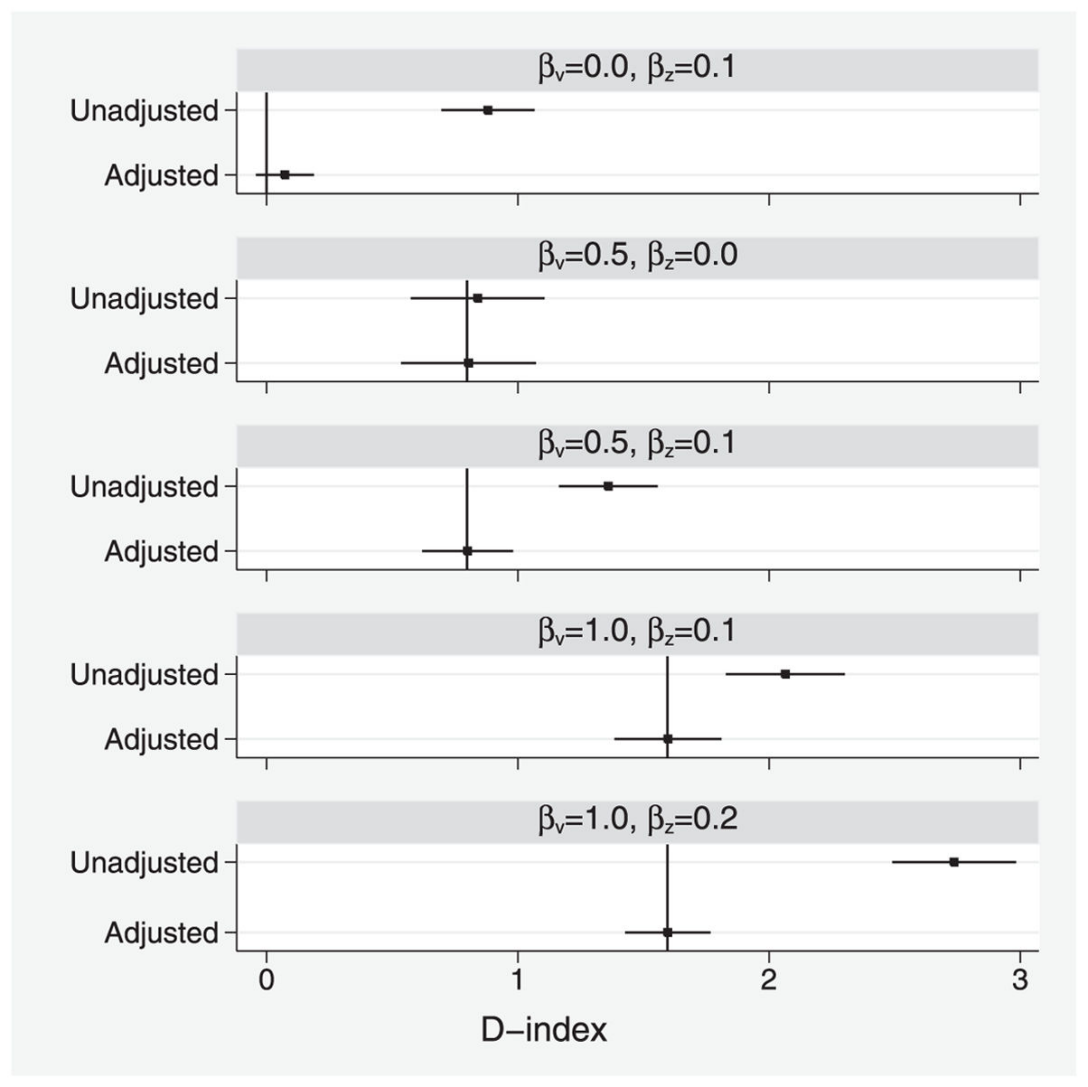
Simulation study 1: comparison of various unadjusted and adjusted estimates of *D*. Intervals show *D̄* ± 1.96*s_D_*. Vertical lines indicate the true value of adjusted *D*.

Results for adjusted *D* similarly suggest small optimism when *β_v_* = 0 and little or no bias elsewhere (Fig. 3).

### 5.5 Results for simulation 2

**Figure 4:**
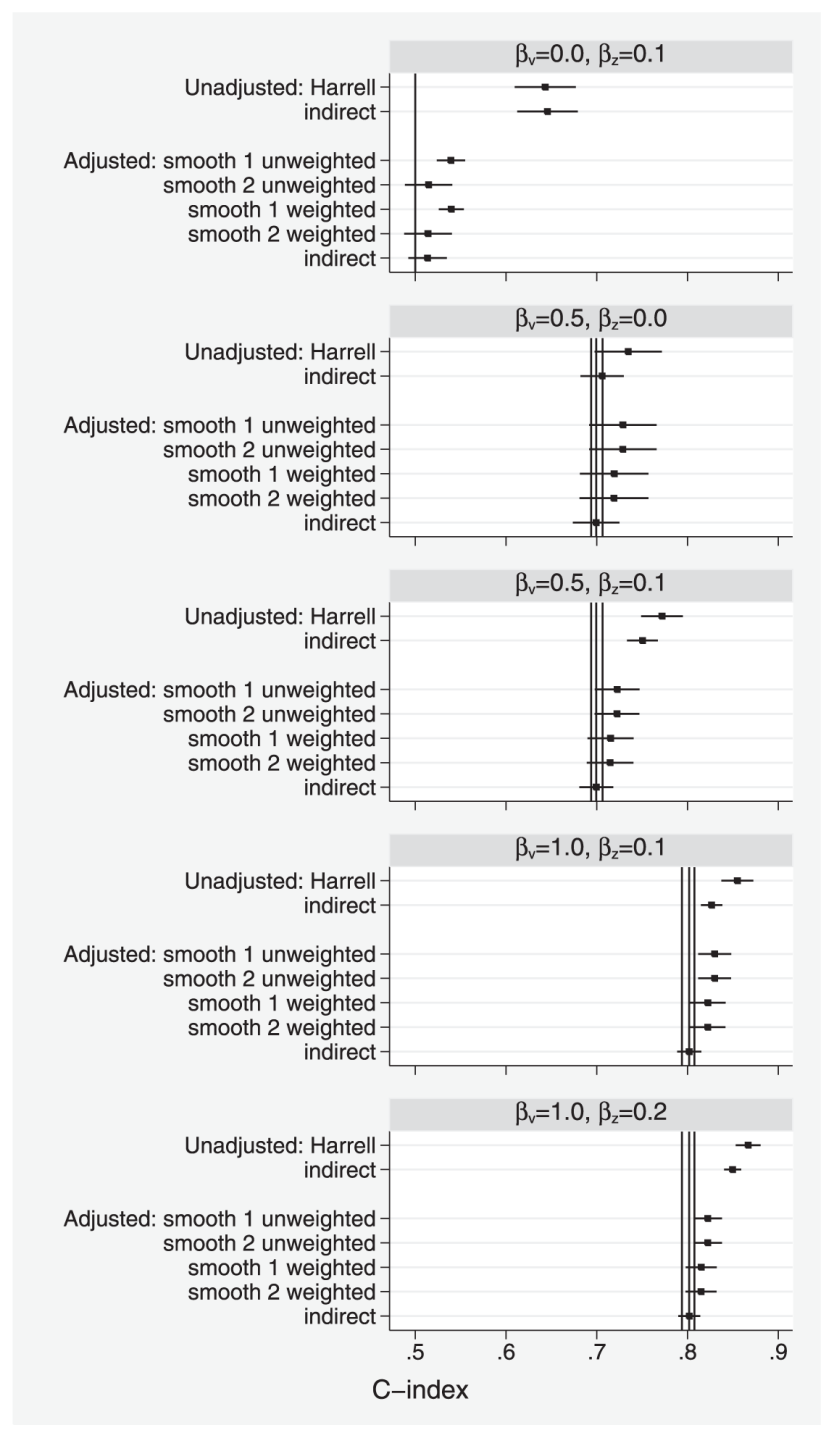
Simulation study 2: comparison of various unadjusted and adjusted estimates of *C*. Vertical lines indicate (from L to R) true values of *C^adj^*^,^*^w^*, C^adj^^*^, and *C^adj^*.

Results for *C* are shown in Fig. 4. When *β_v_* = 0 (top panel), the results are very similar to simulation 1. In the other panels, the estimands *C^adj^* (estimated by the unweighted methods), *C^adj^*^,^*^w^* (estimated by the weighted methods), and C^adj^^*^ (estimated by the indirect method) are unequal and are shown by three vertical lines. These estimands differ by up to 0.014, with *C^adj^*^,^*^w^* < C^adj^^*^ < *C^adj^*.

${\hat{C}}_{\mathrm{ind}}^{\mathrm{adj}}$ remains unbiased for C^adj^^*^. The smoothing estimators are all positively biased, with weighted estimators on average slightly smaller than unweighted estimators. In the second panel, where unadjusted and adjusted C-indices are equal, the bias in the smoothing estimators is slightly smaller than that in Harrell’s estimator: this suggests that all the bias observed is attributable to censoring.

**Figure 5:**
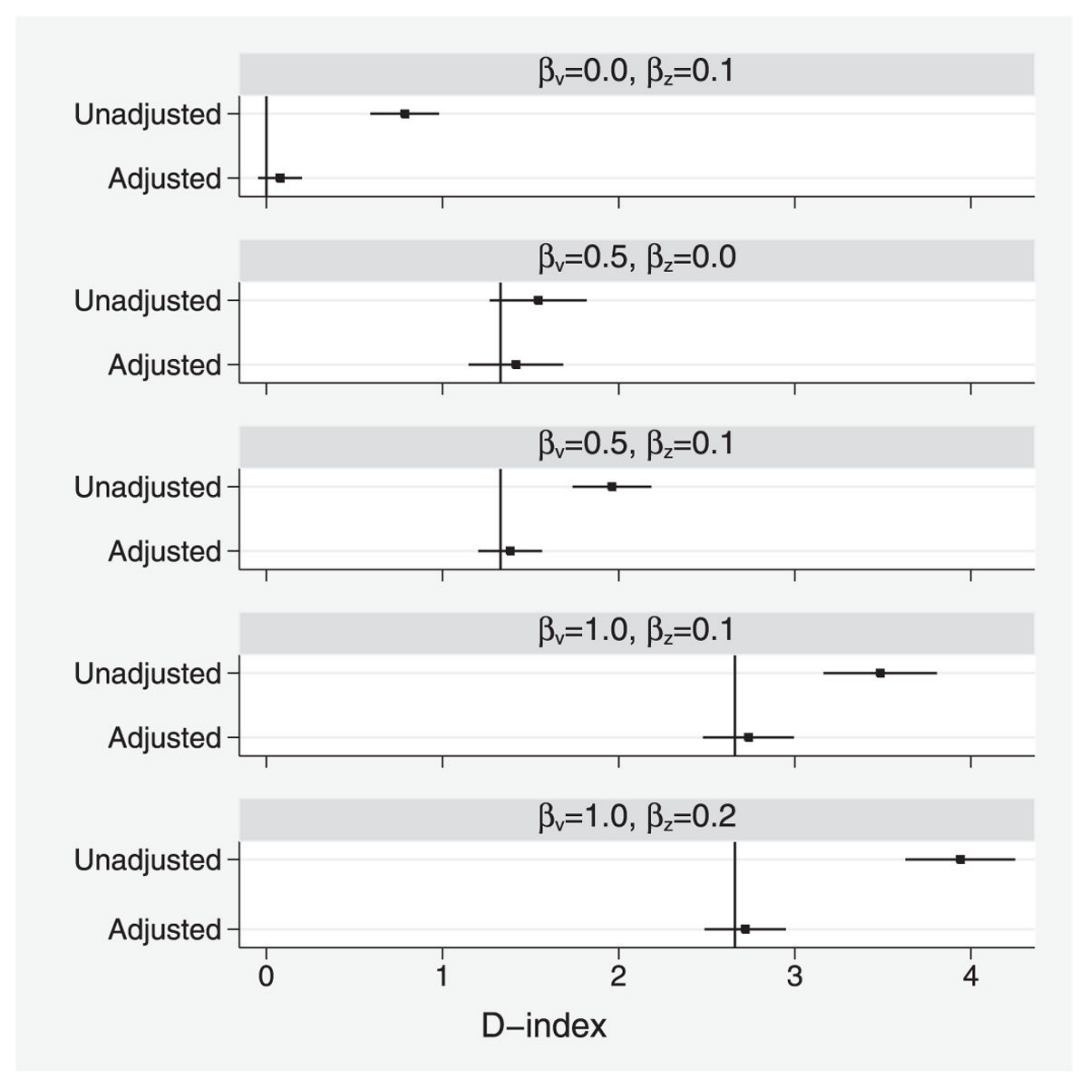
Simulation study 2: comparison of various unadjusted and adjusted estimates of *D*. Vertical lines indicate the true value of adjusted *D*.

Corresponding results for *D* are shown in Fig. 5. Small positive bias is found for all parameter values. Because no bias was seen in simulation 1, this is likely to arise from model mis-specification.

## 6 ERFC results

To illustrate the differences between methods and the effects of recalibration, we used the single Cox proportional hazards model fitted to all the ERFC studies, stratifying by study, sex, and trial arm, as displayed in Table 1. The linear predictor from the resulting model was evaluated using unweighted methods in each study separately, stratifying by sex and trial arm.

**Figure 6:**
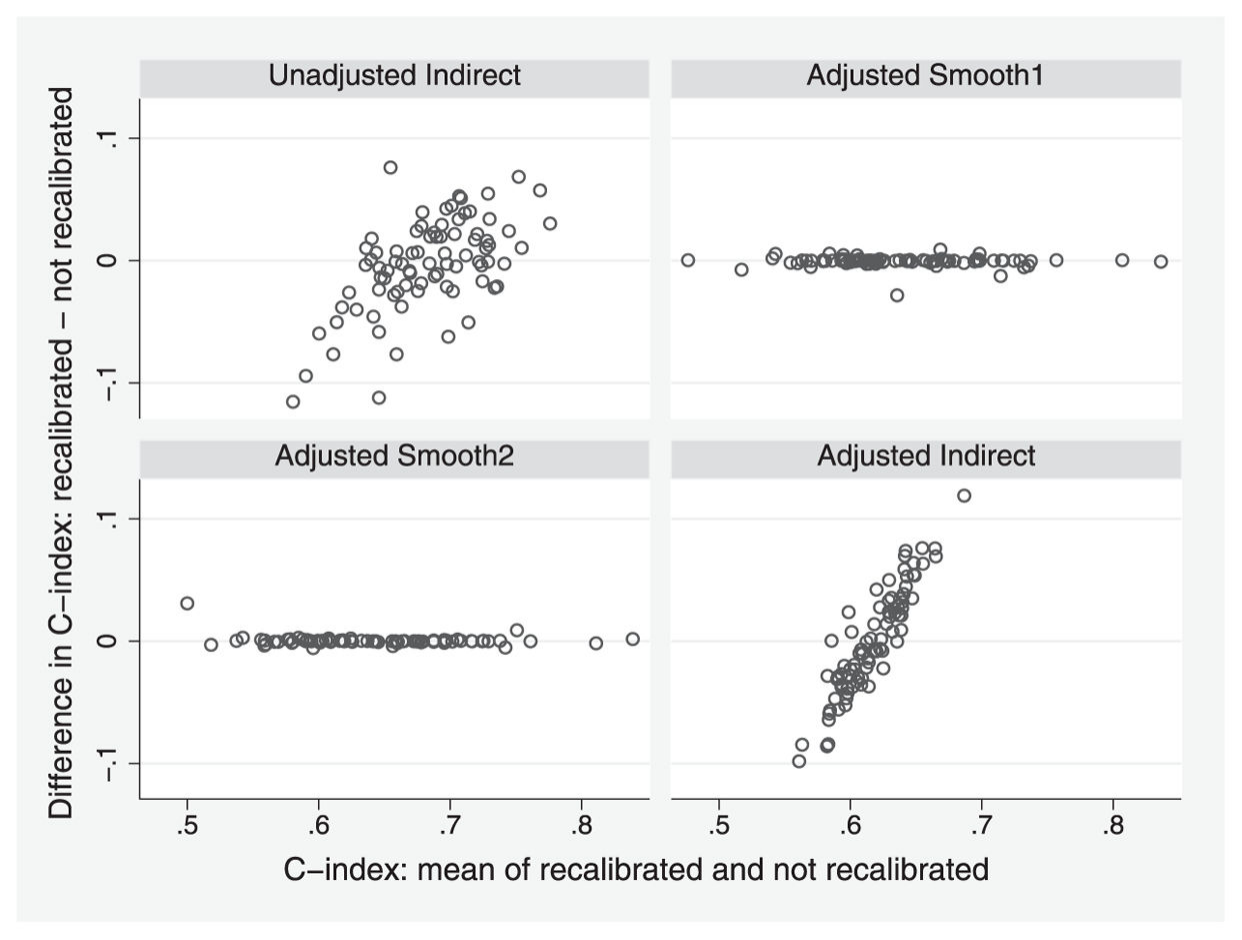
ERFC data: Bland-Altman plots exploring the effects of recalibration on various methods for computing the adjusted C-index. Each point represents one study.

We first explore the effect of recalibration. The model is guaranteed to be well calibrated in the whole ERFC data, but it is likely to be miscalibrated in individual studies. Figure 6 plots, for each method considered, the difference between the C-indices after recalibration and before recalibration against their mean, as proposed by Bland and Altman (1986). Harrell’s method is unaffected by recalibration and so is not shown in Fig. 6. We see that recalibration has a large impact for the indirect methods and very little impact for the smoothing methods.

**Figure 7:**
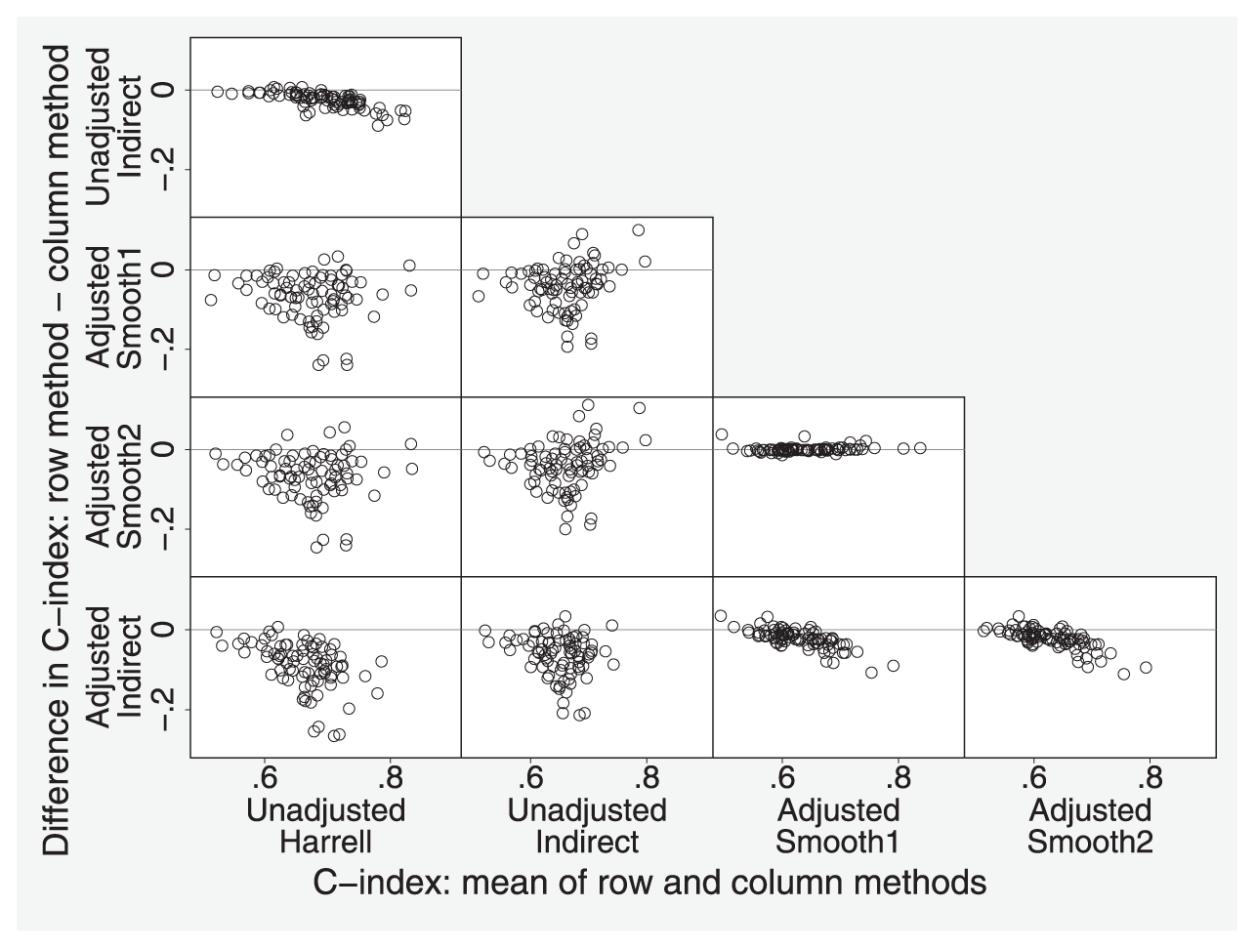
ERFC data: Bland-Altman plots comparing different methods for computing the adjusted C-index, after recalibration. Each point represents one study.

We next compare the different methods after recalibration. Figure 7 shows Bland-Altman plots comparing the two unadjusted methods and the three adjusted methods. The top panel shows that the indirect method tends to give lower results than Harrell’s method, probably due to censoring (Gonen and Heller, 2005). The four panels in the lower left-hand corner compare unadjusted and adjusted methods and show large differences. The three panels in the lower right-hand corner compare the adjusted methods. Again, the indirect method tends to give lower estimates than the other methods, while the two smoothing methods give very similar results.

**Figure 8:**
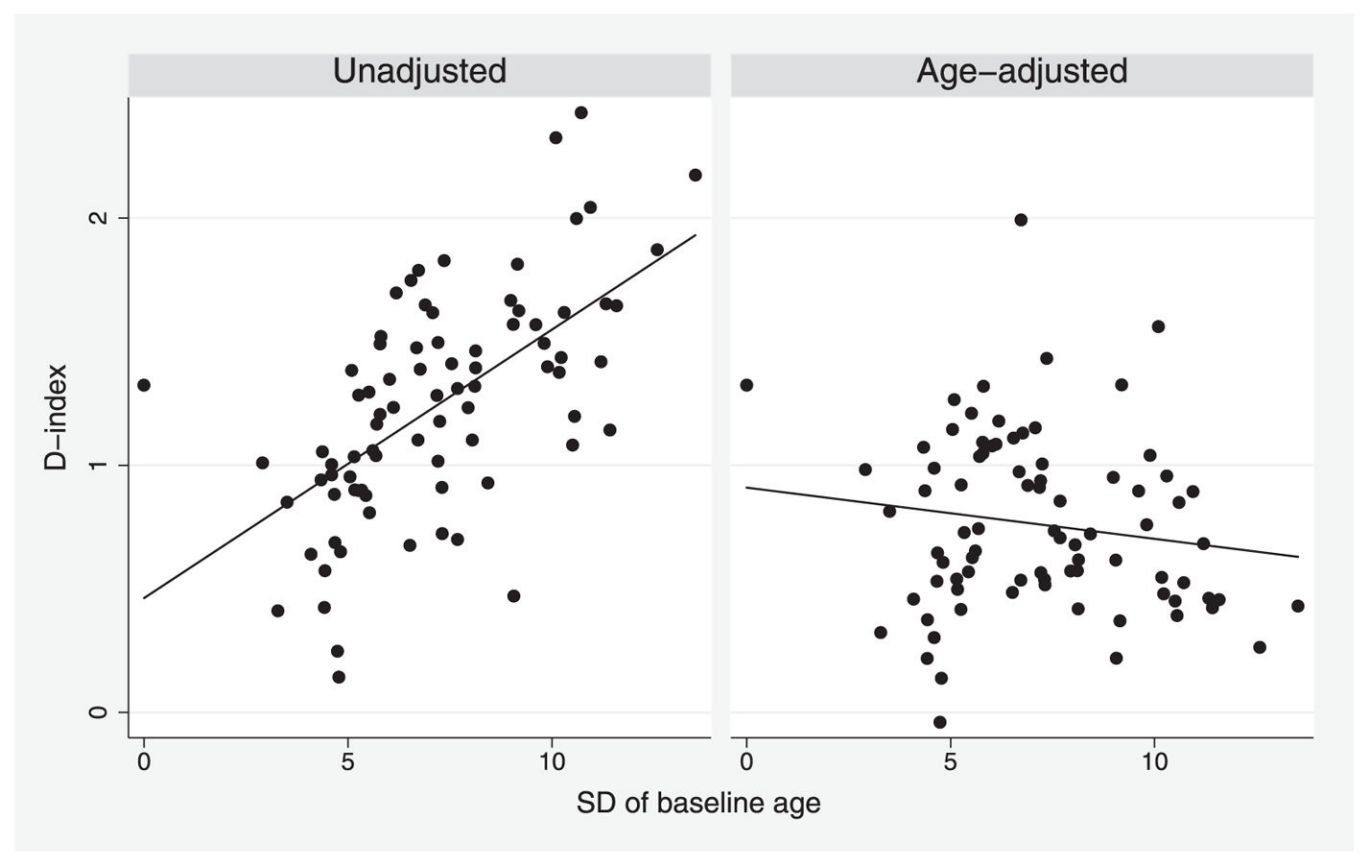
ERFC data: unadjusted and age-adjusted D-index for a model including baseline age, smoking, SBP, TC, and HDL, plotted for each study against the SD of baseline age in that study. Analyses are stratified by sex and trial arm. Each point represents one study.

Finally, we revisit Fig. 1, which used the indirect method with recalibration. The strong association of the unadjusted C-index with SD of baseline age (left-hand panel) is removed when we use the age-adjusted C-index (right-hand panel). Covariate adjustment reduces *C* by up to 0.21 in 77 of the 82 studies; increases *C* by up to 0.03 in four studies (all of which are small); and leaves *C* unchanged for one study where all participants have the same baseline age. Figure 8 shows the corresponding results for the D-index.

Source code to analyze simulated data (like one ERFC cohort) is available as Supporting Information on the journal’s web page (http://onlinelibrary.wiley.com/doi/10.1002/bimj.201400061/suppinfo).

## 7 Discussion

We have proposed a number of methods for estimating an adjusted C-index. The lower part of Table 3 lists a number of desirable properties of an adjusted measure of discrimination, and evaluates the proposed adjusted C-indices and the adjusted D-index against these measures. Overall, the best adjusted C-index appears to be the indirect estimator, although we caution that it is sensitive to model mis-specification. The adjusted D-index is an excellent alternative which is easy to compute.

We have not discussed computation of standard errors in this paper: for the C-index, bootstrapping could be used, while a standard error arises naturally in the calculation of the D-index.

Optimism (overfitting) is an issue whenever models are estimated and evaluated on the same dataset (Harrell et al., 1996). It is not the focus of this paper, because the ERFC data set is large and optimism is likely to be negligible. However, there were signs of optimism in the simulation studies with *β_v_* = 0. In general, our methods should be applied after recalibrating the risk score *r*(**x**) to allow for optimism, ideally in an external validation set, or otherwise by using internal corrections such as the bootstrap (Harrell et al., 1996).

Censoring is another potential problem for our methods. It causes bias in direct methods if noninformative pairs are simply excluded. Our simulation results show that moderate biases due to censoring do occur, especially in larger C-indices (e.g., in the bottom two panels of Fig. 4). Typically, studies have both a limit *τ* to the length of follow-up and random censoring before that time. The method of Uno et al. (2011) can be used to correct for the random censoring, but it estimates *C^τ^* not *C*. Currently the only way to estimate *C* with data censored by end of follow-up is the indirect method.

Model mis-specification is a further potential problem, especially with the indirect methods which assume a correctly specified proportional hazards model. We have proposed a recalibration step which should remove bias due to miscalibration, but not necessarily other forms of model mis-specification. The direct methods such as Smooth 1 and Smooth 2 should be much less sensitive to model mis-specification, since they depend on observed concordance, not model-predicted concordance.

To reduce sensitivity to model mis-specification, we tried using the difference between the direct and indirect estimators of *C* (which may reflect the impact of model mis-specification) to correct the indirect estimator of C^adj^^*^, defining ${\hat{C}}_{\mathrm{corr}1}^{\mathrm{adj}*}={\hat{C}}_{\mathrm{Har}}-{\hat{C}}_{\mathrm{ind}}+{\hat{C}}_{\mathrm{ind}}^{\mathrm{adj}*}$ (or the equivalent on the logit scale). However, because the impact of censoring is greater in unadjusted than adjusted estimators (Fig. 2), the corrected estimator did not perform well in the simulation study and was not included in the results.

Covariate adjustment could be considered for other measures of discrimination. The net reclassification index (NRI) is a popular measure of the difference in discrimination between two models (Pencina et al., 2008). Because the NRI is based on within-individual comparisons, it is neither necessary nor possible to adjust it for covariates, although a covariate-specific NRI could be a useful quantity. However, correct calibration is required to avoid misleading NRI results (Hilden and Gerds, 2014). Another way to evaluate the value of adding a new biomarker to a risk prediction model could be to evaluate the discrimination of the new model, adjusting for the covariates in the original model.

Further extensions include ways to account for competing risks and to obtain time-dependent measures of discrimination (Wolbers et al., 2009). A common feature of these approaches is that the C-index needs to be computed for different time points which limit the comparability of different studies that have different length of follow-up. An open question is which metric is more appropriate for which data and whether these different approaches can produce different conclusions in some scenarios. The methodology presented here could be extended to incorporate these extensions.

Our approach should not be confused with ROC regression (Tosteson and Begg, 1988). ROC regression methods model the accuracy of a diagnostic test as a function of covariates, not how the disease is associated with covariates. A possible use of such an approach might be to find subgroups where the marker should not be used or to find optimal cutoffs. Here we assume predictions that have been optimized with respect to disease risk and our aim is not (primarily) to explain which covariates affect accuracy but to adjust discrimination statistics for the confounding effect of the covariates that do.

In summary, we have proposed covariate-adjusted measures of concordance. There are many benefits to such measures (Pepe et al., 2008). In the meta-analysis setting, they facilitate comparisons between studies with different covariate distributions (Figs. 1 and 8). They also enable matched case-control studies nested within cohort studies to be compared with standard cohort studies, since the former can only yield measures of discrimination adjusted for the matching variables. We advocate adjustment, at least for study design variables such as age, sex, and study centre, whenever measures of discrimination are to be compared between studies with different distributions of the design variables.

## Figures and Tables

**Table 1:** ERFC data: variable summaries and selected model.

Variable	Mean	Within-	Between-	Fitted model	
	
		studies SD	studies SD	Coef.	Std. Err.
Baseline variables					
Age (years)	55.92	7.57	6.78	0.0771	0.0009
Smoking (0 = no, 1 = yes)	0.30	0.43	0.15	0.563	0.014
SBP (mm Hg)	133.30	18.51	7.70	0.0146	0.0003
Total cholesterol (mmol/L)	5.87	1.07	0.46	0.168	0.006
HDL cholesterol (mmol/L)	1.35	0.37	0.15	−0.512	0.020
Sex (0 = male, 1 = female)	0.42	0.40	0.29	(stratifier)	
Outcome variables					
Follow-up (years)	10.64	3.65	4.87		
CVD event (0 = no, 1 = yes)	0.07	0.24	0.07		

**Table 2:** Summary of methods for unadjusted measures of discrimination.

	C-index		D-index
	*Harrell’s C*	*Indirect*	
Notation	*Ĉ_Har_*	*Ĉ_ind_*	*D̂*
Description	Mean concordance among informative pairs	Mean expected concordancea)	Cox model on scaled rankit of risk score
Quantity estimated	*C*	*C*	*D*
Recalibration	No impact	Needed	Implicit in method

a) Expected concordance is computed assuming that *r*(**x**) is the linear predictor in a correctly specified Cox model.

**Table 3:** Summary of methods for adjusted measures of discrimination.

	C-index			Adjusted D-index
	Smooth 1	Smooth 2	Indirect	
Notation	${\hat{C}}_{\mathrm{smooth}1}^{\mathrm{adj}},{\hat{C}}_{\mathrm{smooth}1}^{\mathrm{adj},w}$	${\hat{C}}_{\mathrm{smooth}2}^{\mathrm{adj}},{\hat{C}}_{\mathrm{smooth}2}^{\mathrm{adj},w}$	${\hat{C}}_{\mathrm{ind}}^{\mathrm{adj}}$	*D^adj^*
Description	Mean concordance (Smooth 1) or *m*-concordance (Smooth 2) among informative pairs with similar values of adjustment variable	Mean expected concordancea) after removing difference in adjustment variable	Covariate-adjusted Cox model on scaled rankit of risk score	
Options	Choice of weights; choice of smoothing parameter *λ*	–	–	
Quantity estimated	*C^adj^* or *C^adjw^*	C^adj^^*^	*D^adj^*	
Recalibration	Little impact	Little impact	Needed	Implicit in method
Properties				
Must lie between 0 and 1	✓	✓	✓	N/A
Has value 1 if all pairs are concordant	✓	✓	✕	N/A
Has value 0 if all pairs are discordant	✓	✓	✕	N/A
Reduces to unadjusted estimate if there is no *Z*	✓	✓	✓	✓
Direct – unaffected by miscalibration	✓	✓	✕	(✕)
Free of tuning parameter *λ*	✕	✕	✓	✓
Fast to compute	✕	✕	✕	✓
Unaffected by optimism	✕	✕	✕	✕
Unaffected by censoring	✕	✕	✓	✓
Unbiased in simulation after accounting for optimism and censoring	✕	✓	✓	✓

a) Expected concordance is computed assuming that *r*(**x**) is the linear predictor in a correctly specified Cox model. (✕) means only slightly affected.

## Appendix

### A Members of the Emerging Risk Factors Collaboration

**Emerging Risk Factors Collaboration investigators/contributors: AFTCAPS:** R W Tipping; **ALLHAT:** B R Davis, L M Simpson; **ARIC:** C M Ballantyne, A R Folsom, J Coresh; **AUSDIAB:** J E Shaw, R Atkins, P Z Zimmet, E L M Barr; **BHS:** M W Knuiman; **BRHS:** S G Wannamethee, R W Morris; **BRUN:** J Willeit, P Willeit, P Santer, S Kiechl; **BUPA:** N Wald; **BWHHS:** S Ebrahim, D A Lawlor; **CAPS:** J Gallacher, J W G Yarnell, Y Ben-Shlomo; **CASTEL:** E Casiglia, V Tikhonoff; CHARL; S E Sutherland, P J Nietert, J E Keil, D L Bachman; **CHS:** B M Psaty, M Cushman, see http://www.chsnhlbi.org for acknowledgments; **COPEN:** B G Nordestgaard, A Tybjærg-Hansen, R Frikke-Schmidt; **CUORE:** S Giampaoli, L Palmieri, S Panico, L Pilotto, D Vanuzzo; **DUBBO:** L A Simons, Y Friedlander, J McCallum; **EAS:** J F Price, S McLachlan; **EPESEBOS:** J O Taylor, J M Guralnik; **EPESEIOW:** R B Wallace, F J Kohout, J C Cornoni-Huntley, J M Guralnik; **EPESENCA:** D G Blazer, J M Guralnik, C L Phillips; **EPESENHA:** C L Phillips, J M Guralnik; **EPICNOR:** N J Wareham, K-T Khaw; **ESTHER:** H Brenner, B Schöttker, H T Müller, D Rothenbacher; **FINE_FIN:** A Nissinen; **FINE_IT:** C Donfrancesco, S Giampaoli; **FINRISK92, FINRISK97:** K Harald, P R Jousilahti, E Vartiainen, V Salomaa; **FRAMOFF:** R B D’Agostino Sr., P A Wolf, R S Vasan; **FUNAGATA:** M Daimon, T Oizumi, T Kayama, T Kato; **GOH:** A Chetrit, R Dankner, F Lubin; **GOTO43:** L Welin, K Svärdsudd, H Eriksson, G Lappas; **GOTOW:** L Lissner, K Mehlig, C Björkelund; **GRIPS:** D Nagel; **HISAYAMA:**Y Kiyohara, H Arima, T Ninomiya, J Hata; **HONOL:** B Rodriguez; **HOORN:** J M Dekker, G Nijpels, C D A Stehouwer; **IKNS:** H Iso, A Kitamura, K Yamagishi, H Noda; **ISRAEL:** U Goldbourt; **KIHD:** J Kauhanen, J T Salonen, T-P Tuomainen; **LEADER:** T W Meade, B L DeStavola; **MCVDRFP:** A Blokstra, W M M Verschuren; **MESA:** M Cushman, I H de Boer, A R Folsom, B M Psaty, see http://www.mesa-nhlbi.org for acknowledgements; **MOGERAUG1, MOGERAUG2:** W Koenig, C Meisinger, A Peters; **MORGEN:** W M M Verschuren, H B Bueno-de-Mesquita, A Blokstra; **MOSWEGOT:** A Rosengren, L Wilhelmsen, G Lappas; **MRFIT:** L H Kuller, G Grandits; **NHANESIII; NPHSII:** J A Cooper, K A Bauer; **NSHS:** K W Davidson, S Kirkland, J A Shaffer, D Shimbo; **OSAKA:** A Kitamura, H Iso, S Sato; **PREVEND:** R P F Dullaart, S J L Bakker, R T Gansevoort; **PRIME:** P Ducimetiere, P Amouyel, D Arveiler, A Evans, J Ferrières; **PROCAM:** H Schulte, G Assmann; PROSPER; J W Jukema, R G J Westendorp, N Sattar; **QUEBEC:** B Cantin, B Lamarche, J-P Després; **RANCHO:** E Barrett-Connor, D L Wingard, L B Daniels; **REYK:** V Gudnason, T Aspelund; **RIFLE:** M Trevisan; **ROTT:** A Hofman, O H Franco; **SHHEC:** H Tunstall-Pedoe, R Tavendale, G D O Lowe, M Woodward; **SHS:** W J Howard, B V Howard, Y Zhang, L G Best, J Umans; **SPEED:** Y Ben-Shlomo, G Davey-Smith; **TARFS:** A Onat; **TOYAMA:** H Nakagawa, M Sakurai, K Nakamura, Y Morikawa; **TROMSO:** I Njølstad, E B Mathiesen, T Wilsgaard; **ULSAM:** J Sundström; **USPHS2:** J M Gaziano, P M Ridker; **WHITE1:** M Marmot, R Clarke, R Collins, A Fletcher; **WHITE2:** E Brunner, M Shipley, M Kivimaki; **WHS:** P M Ridker, J Buring, N Rifai, N Cook; **WOSCOPS:** I Ford, M Robertson; **ZARAGOZA:** A Marín Ibañez; **ZUTE:** E J M Feskens, J M Geleijnse.

**Coordinating Centre**: T Bolton, S Burgess, A S Butterworth, E di Angelantonio, P Gao, E Harshfield, S Kaptoge, L Pennells, S Peters, S Spackman, S Thompson, M Walker, I White, P Willeit, A Wood, J Danesh (Principal Investigator).

### B Comparison of estimands

We give an example where var(*V*|*Z* = *z*) does not depend on *z* and yet the estimands *C^adj^*^*^ and*C^adj^* are unequal.

Suppose **X** = (*V*, *Z*) where *V* and *Z* are binary with *p*(*Z* = 1) = 0.5, *p*(*V* = 1|*Z* = 0) = *π*_0_ = 0.2 and *p*(*V* = 1|*Z* = 1) = *π*_1_ = 0.8. Suppose *h_i_*(*t*) = *h*_0_(*t*) exp(*V* + *Z*) so *r*(**X**) = *V* + *Z*. We want to adjust for *Z* so *m*(**X**, *Z*) = *V* − E[*V*|*Z*]. When *Z_i_* = *Z_j_* = *z*, |*m*(**X***_i_*, *Z_i_*) − *m*(**X***_j_*, *Z_j_*)| is 1 with probability 2*π_z_*(1 − *π_z_*) and 0 otherwise, and this distribution is independent of *z* since *π*_1_ = 1 − *π*_0_. So *C*(*z* = 0) = *C*(*z* = 1) = *C^adj^* = 0.574. But for pairs with *Z_i_* ≠ *Z_j_*, |*m*(**X***_i_*, *Z_i_*) − *m*(**X***_j_*, *Z_j_*)| has a different distribution (but the same SD). As a result, *C^adj^*^*^ = 0.598 ≠ *C^adj^*.

### C True values of *C*(*z*), *C^adj^,* and *C^adj^*^*^ in the simulation study

For simplicity we assume *β_v_* ≥ 0. We also assume that the model is correctly specified. We first compute *C*(*z*), for which the distribution of *z* is not needed. Define *I*(*σ*^2^) = E[expit {|*A*|}] when *A* ~ *N*(0, *σ*^2^). Then

$$I(\sigma^{2})=2\int_{0}^{\infty }\text{expit}\;(\sigma u)\;\phi \;(u)du$$

where *ϕ*(*u*) is the standard normal density function.

For two individuals *r* = 1, 2 with *z*_1_ = *z*_2_ = *z*, we can write *v_r_* = *α*(*z* − 50) + *u_r_*, so the probability that they are concordant is expit(|*β_v_*(*v*_2_ − *v*_1_)|) = expit(|*β_v_*(*u*_2_ − *u*_1_)|). If *z* is in age group *g* then $u_{r}\sim N(0,\sigma_{g}^{2})$ so $\mathrm{var}(\beta_{v}(u_{2}-u_{1}))=2\beta_{v}^{2}\sigma_{g}^{2}$ and

(A.1) $$C(z)=I(2\beta_{v}^{2}\sigma_{g}^{2}).$$

For simulation 1, we evaluate (A.1) with *σ_g_* = 1. We then have *C^adj^* = *C^adj^*^*^ = *C*(*z*) for all *z*.

For simulation 2, *σ*_1_ = 1 and *σ*_2_ = 2. Denote the corresponding values of *C*(*z*) from (A.1) as $C_{1}=I(2\beta_{v}^{2}\sigma_{1}^{2})$ and $C_{2}=I(2\beta_{v}^{2}\sigma_{2}^{2})$. We can then derive *C^adj^* = {(1 − *ϕ*)^2^*C*_1_ + *ϕ*^2^*C*_2_}/{(1 − *ϕ*)^2^ + *ϕ*^2^} and *C^adj^*^,^*^w^* = (1 − *ϕ*)*C*_1_ + *ϕC*_2_. Finally, we can write *C^adj^*^*^ as a weighted sum of E[expit {*β_v_*|*u*_1_ − *u*_2_|}] terms over the possible groups for individuals 1 and 2:

$$C^{\mathrm{adj}*}={\left(1-\phi \right)}^{2}I(2\beta_{v}^{2}\sigma_{1}^{2})+2\phi (1-\phi )I(\beta_{v}^{2}(\sigma_{1}^{2}+\sigma_{2}^{2}))+{\left(1-\phi \right)}^{2}I(2\beta_{v}^{2}\sigma_{2}^{2}).$$

### D True value of *D^adj^*

We have *D* = E[*r*(**x***_i_*) − *r*(**x***_j_*)|*z_i_* = *z_j_*, *m*(**x***_i_*, *z_i_*) < 0 < *m*(**x***_j_*, *z_j_*)]. We again assume that the model is correctly specified. Since *m*(**x***_i_*, *z_i_*) = *β_v_u_i_*, *D* = *β_v_*{E[*u_i_*|*u_i_* > 0] − E[*u_i_*|*u_i_* < 0]}. In simulation 1, $E[u_{i}|u_{i}>0]=\sqrt{2/\pi }$. In simulation 2, $E[u_{i}|u_{i}>0]=\sqrt{2/\pi }\{(1-\phi )\sigma_{1}+\phi \sigma_{2}\}=\frac{5}{3}\sqrt{\frac{2}{\pi }}$. In both cases, E[*u_i_*|*u_i_* < 0] = −E[*u_i_*|*u_i_* > 0]. Hence $D^{\mathrm{adj}}=\beta_{v}\sqrt{8/\pi }$ in simulation 1 and $D^{\mathrm{adj}}=\beta_{v}\frac{5}{3}\sqrt{8/\pi }$ in simulation 2.
